# Deciphering the Non-Coding RNA Landscape of Pediatric Acute Myeloid Leukemia

**DOI:** 10.3390/cancers14092098

**Published:** 2022-04-22

**Authors:** Jolien Vanhooren, Laurens Van Camp, Barbara Depreter, Martijn de Jong, Anne Uyttebroeck, An Van Damme, Laurence Dedeken, Marie-Françoise Dresse, Jutte van der Werff ten Bosch, Mattias Hofmans, Jan Philippé, Barbara De Moerloose, Tim Lammens

**Affiliations:** 1 Department of Pediatric Hematology-Oncology and Stem Cell Transplantation, Ghent University Hospital, 9000 Ghent, Belgium; laurens.vancamp@ugent.be (L.V.C.); martijn.dejong@ugent.be (M.d.J.); barbara.demoerloose@uzgent.be (B.D.M.); tim.lammens@ugent.be (T.L.); 2 Department of Internal Medicine and Pediatrics, Ghent University, 9000 Ghent, Belgium; 3 Cancer Research Institute Ghent (CRIG), 9000 Ghent, Belgium; mattias.hofmans@uzgent.be (M.H.); jan.philippe@uzgent.be (J.P.); 4 Department of Laboratory Hematology, Vrije Universiteit Brussel (VUB), Universitair Ziekenhuis Brussel, 1050 Brussels, Belgium; barbara.depreter@uzbrussel.be; 5 Department of Pediatrics, University Hospital Gasthuisberg, 3000 Leuven, Belgium; anne.uyttebroeck@uzleuven.be; 6 Department of Pediatric Hematology Oncology, University Hospital Saint-Luc, 1200 Brussels, Belgium; an.vandamme@uclouvain.be; 7 Department of Pediatric Hematology Oncology, Queen Fabiola Children’s University Hospital, 1020 Brussels, Belgium; laurence.dedeken@huderf.be; 8 Department of Pediatric Hematology Oncology, University Hospital Liège, 4000 Liège, Belgium; mf.dresse@chuliege.be; 9 Department of Pediatric Hematology Oncology, University Hospital Brussel, 1090 Brussels, Belgium; jutte01@gmail.com; 10 Department of Diagnostic Sciences, Ghent University, 9000 Ghent, Belgium

**Keywords:** pediatric AML, non-coding RNA, cellular subpopulations

## Abstract

**Simple Summary:**

Survival rates for children with acute myeloid leukemia have significantly improved in recent decades. Still, 20–30% will succumb to therapy-related toxicity and relapse. Research has shown that leukemic stem cells (LSCs) in particular are responsible for relapse. Therefore, the characterization of the LSC is needed to improve the current treatment options for and survival of these pediatric patients. Previously, research has focused mainly on the protein-coding part of the human genome, but recently the focus has also shifted to non-coding genes, including long non-coding RNA (lncRNA) and microRNA (miRNA). The aim of this study was to investigate the expression of lncRNAs and miRNAs in pediatric AML subpopulations (LSCs and leukemic blasts) and their normal counterparts (hematopoietic stem cells and control myeloblasts). We provide here a unique set of non-coding RNAs with a potential role in the pathogenesis of pediatric AML, paving the way for further translational research studies.

**Abstract:**

Pediatric acute myeloid leukemia (pedAML) is a heterogeneous blood cancer that affects children. Although survival rates have significantly improved over the past few decades, 20–30% of children will succumb due to treatment-related toxicity or relapse. The molecular characterization of the leukemic stem cell, shown to be responsible for relapse, is needed to improve treatment options and survival. Recently, it has become clear that non-coding RNAs, including long non-coding RNAs (lncRNAs) and microRNAs (miRNAs), play a role in the development of human diseases, including pediatric cancer. Nevertheless, non-coding RNA expression data in pedAML are scarce. Here, we explored lncRNA (*n* = 30,168) and miRNA (*n* = 627) expression in pedAML subpopulations (leukemic stem cells (LSCs) and leukemic blasts (L-blasts)) and their normal counterparts (hematopoietic stem cells and control myeloblasts). The potential regulatory activity of differentially expressed lncRNAs in LSCs (unique or shared with the L-blast comparison) on miRNAs was assessed. Moreover, pre-ranked gene set enrichment analyses of (anti-) correlated protein-coding genes were performed to predict the functional relevance of the differentially upregulated lncRNAs in LSCs (unique or shared with the L-blast comparison). In conclusion, this study provides a catalog of non-coding RNAs with a potential role in the pathogenesis of pedAML, paving the way for further translational research studies.

## 1. Introduction

Pediatric acute myeloid leukemia (pedAML) is a rare hematological disease that accounts for 20% of all pediatric leukemias [1]. With current chemotherapeutic regimens, the five-year survival rate has reached a plateau of 70% [2,3]. Unfortunately, 30% to 40% of the good responders will experience relapse, often correlated with specific genetic subgroups [2]. The high relapse rate is thought to be caused by the persistence of leukemic stem cells (LSCs). Therefore, therapies that not only target the rapidly dividing leukemic blasts (L-blasts) but also the LSCs are needed to improve the outcomes.

Efforts to characterize alterations in the transcriptional program of the disease have led to the discovery of novel AML-specific targets, albeit mostly focused on the protein-coding part of the transcriptome [4,5,6,7]. The identification of AML-specific therapeutic targets is challenging as co-expression in the hematopoietic stem cell (HSC) is often observed, leading to severe cytopenia when targeted [8]. As AML is a biologically complex disease, dictated in part by mutational, clonal, and epigenetic heterogeneity, the knowledge on its pathogenesis is still evolving [9]. Recently, an LSC-specific signature of 111 lncRNAs characterizing cytogenetic normal adult AML was uncovered, revealing potentially interesting novel targets for therapy [10]. The role and function of non-coding transcripts are being increasingly explored, further expanding our knowledge on the pathogenesis of pedAML and providing therapeutic and diagnostic opportunities. Long non-coding RNAs (lncRNAs), non-coding transcripts of >200 nucleotides, constitute a heterogeneous group of non-coding RNAs acting as key regulators of gene transcription, protein translation, and epigenetic regulation [11]. Interestingly, compared to messenger RNAs, the expression of lncRNAs seems to be much more tissue specific [12,13], making them highly interesting therapeutic targets. Research has shown that lncRNAs can harbor binding sites for miRNAs—non-coding transcripts of approximately 18–25 nucleotides that negatively regulate gene expression post-transcriptionally—acting as endogenous competitors [14,15,16,17]. Subsequently, lncRNAs can sequester or sponge miRNAs, resulting in their dysregulated activity on physiologically relevant target genes. The process of sponging has been widely studied in diverse cancer entities [18], including AML.

Here, we characterized the lncRNA expression in the LSCs and L-blasts in pedAML and identified key differentially expressed (DE) lncRNAs compared to their respective healthy counterparts, taking into account a threshold for their expression in the HSCs. To unravel potential endogenous competitors, miRNA-lncRNA networks of anti-correlated miRNAs to the identified DE-lncRNAs were examined. Furthermore, a gene set enrichment analysis (GSEA) was performed to uncover associated pathways. Altogether, our results highlight the role of lncRNAs in the pathogenesis of pedAML and contribute to deciphering the biological complexity of the disease. To this end, we identified key non-coding RNAs that need further evaluation regarding their roles as novel biomarkers for risk stratification, follow-up, and targeted therapy.

## 2. Materials and Methods

### 2.1. Patients and Controls

Bone marrow (BM) and/or peripheral blood (PB) from four pedAML patients with diverse molecular characteristics was selected based on cell availability (>50 × 10^6^ after routine workup) and CD34+ positivity (≥1%) (Table 1). As a control, cord blood (CB = 3) obtained after full-time delivery was used. All subjects gave their informed consent for inclusion before participation. The study was conducted in accordance with the Declaration of Helsinki, and the protocol was approved by the Ethics Committee of the University Hospital of Ghent (EC2015-1443 and EC2019-0294).

### 2.2. Cell Sorting

Mononuclear cells were isolated by Ficoll density gradient (Axis-Shield Diagnostics Ltd., Dundee, Scotland), complemented by CD34 isolation if the CD34 expression was <50% (CD34 MicroBead Kit, Milteny Biotec B.V., Leiden, The Netherlands). Cell sorting was performed to isolate CD34+/CD38− and CD34+/CD38+ cells from patients and controls, defined as LSCs and HSCs, and L-blasts and control blasts (C-blasts), respectively. Sorting was performed according to the method of Depreter and colleagues [19], and a detailed description can be found in the Supplemental Material and Methods. Sorted cells were collected in RPMI supplemented with 50% FCS, and a post-sort purity of >90% was reached. Antibodies used for sorting are shown in Table S1. Sorted cells were spun down (10 min, 3000 rpm, 4 °C) and resuspended in 700 μL of TRIzol for RNA extraction.

### 2.3. RNA Isolation

RNA was extracted using the miRNeasy Mini or Micro Kit (QIAGEN Benelux B.V. —Belgium, Antwerp, Belgium) in combination with on-column DNase I digestion (RNAse-Free DNase set, Qiagen) according to the manufacturer’s instructions. RNA concentrations were measured by Nanodrop (ThermoFisher Scientific BVBA, Merelbeke, Belgium) or Qubit RNA HS Assay (Invitrogen, Merelbeke, Belgium).

### 2.4. Micro-Array Analysis

Three LSC and four L-blast fractions, next to two HSCs and three C-blast controls, were profiled on a custom-designed Agilent 8 × 60 K humane gene expression micro-array platform by Biogazelle. This micro-array contained probes for all human protein-coding genes (*n* = 27,071) and lncRNA probes (*n* = 30,168) based on the LNCipedia database for lncRNAs [20]. Micro-array profiling was performed according to Depreter et al. [19], and details can be found in Supplementary Methods. Differentially expressed (DE) lncRNAs were identified based on |log2FC| > 2 and adjusted *p*-values (adj. *p*) ≤ 0.05 using the limma package (R Bioconductor). However, if a differentially expressed gene was represented by multiple probes with an equal transcript, the one with the highest fold change was selected for further downstream analyses. Raw data can be found under GSE 128,103 (released on 9 March 2022).

### 2.5. Small RNA-Sequencing Analysis

Small RNA expression profiling was performed using Biogazelle on the same sample cohort. Libraries for RNA sequencing were prepared using Biogazelle and a TruSeq small RNA library kit (Illumina) according to the manufacturer’s instructions. The resulting small RNA libraries were concentrated via ethanol precipitation and quantified using the Qubit 2.0 Fluorometer before sequencing with a read length of 75 bp on a NextSeq 500 sequencer (Illumina). Mapped reads were subsequently annotated to mature miRNAs using genome annotation data from Ensemble release 84, UCSC, and miRbase v21. Prior to normalization, data were filtered using a cut-off of 4 reads. miRNA expression data were normalized based on the total read count per sample. After normalization, a pseudo-count of 1 to each read miRNA count was added before log transformation. DE-miRNAs were examined based on |log2FC| > 2 and adj. *p* ≤ 0.05 using the EdgeR and limma packages (R Bioconductor). Raw data can be found under GSE 196,886 (released on 16 August 2022).

### 2.6. miRNA-lncRNA Network Constuction

Pearson correlations between miRNAs and LSC DE-lncRNAs as miRNAs and shared DE-lncRNAs were computed to construct a miRNA-lncRNA network, using the base R *cor()* function. The threshold for anti-correlated miRNAs was set at a Pearson correlation coefficient of −0.7.

### 2.7. Gene Set Enrichment Analysis

A Pearson correlation matrix was computed between the unique upregulated LSC DE-lncRNAs, as well as the shared DE-lncRNAs, and a set of protein-coding genes (*n* = 27,071) using the base R *cor()* function. For every (anti-)correlated (|ρ| > 0.7) protein-coding gene, the mean of the absolute expression values in the four fractions (LSC, L-blast, HSC, and C-blast) was calculated. A pre-ranked gene set enrichment analysis (GSEA) was performed using GSEA 4.1.0 (by Broad Institute) for the respective (anti-)correlated protein-coding genes of each selected DE-lncRNA to explore pathways of involvement using the hallmark gene set (*n* = 50) (www.gsea-msigdb.org, accessed on 22 December 2021) The HGNC ID nomenclature was used as a chip platform for the GSEA. The false discovery rate (FDR) was set at <10%. The genes *TAF9* and *HNRNPA2B1* were removed from the enrichment analysis, as the different probes were both correlated and anti-correlated to the respective DE-lncRNA, resulting in conflicting results.

## 3. Results

### 3.1. Identification of Differentially Expressed lncRNAs in pedAML Subpopulations

**Figure 1:**
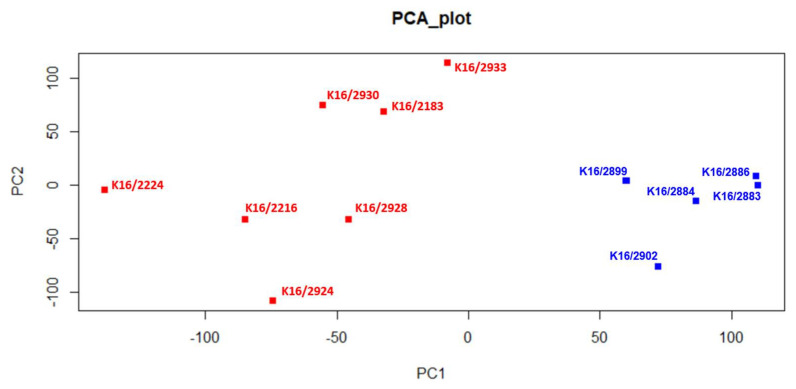
Principal component analysis (PCA). PedAML samples and healthy CB samples are colored in red and blue, respectively. Samples K16/2886 and K16/2883 refer to CD34+/CD38− fractions isolated from healthy CB. Samples K16/2899, K16/2884, and K16/2902 refer to CD34+/CD38+ fractions isolated from healthy CB. Samples K16/2924, K16/2933, and K16/2930 refer to CD34+/CD38− fractions isolated from pedAML patients. Samples K16/2224, K16/2216, K16/2928, and K16/2183 refer to CD34+/CD38+ fractions isolated from pedAML patients.

**Figure 2:**
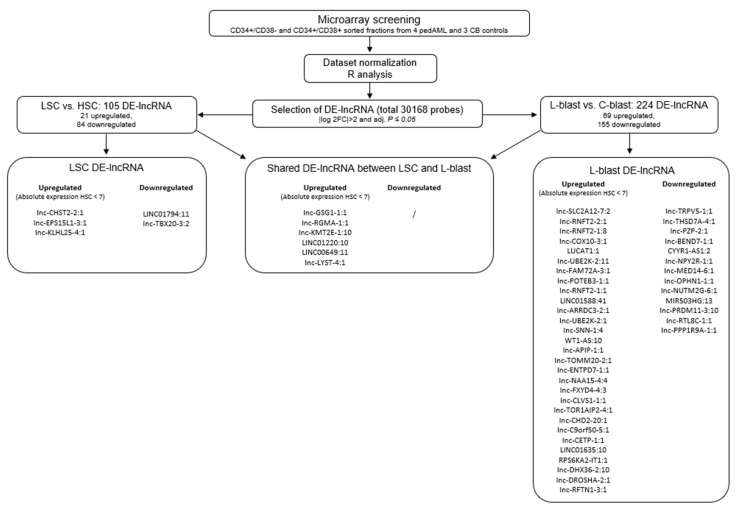
Workflow and selection process of the micro-array analysis.

**Figure 3:**
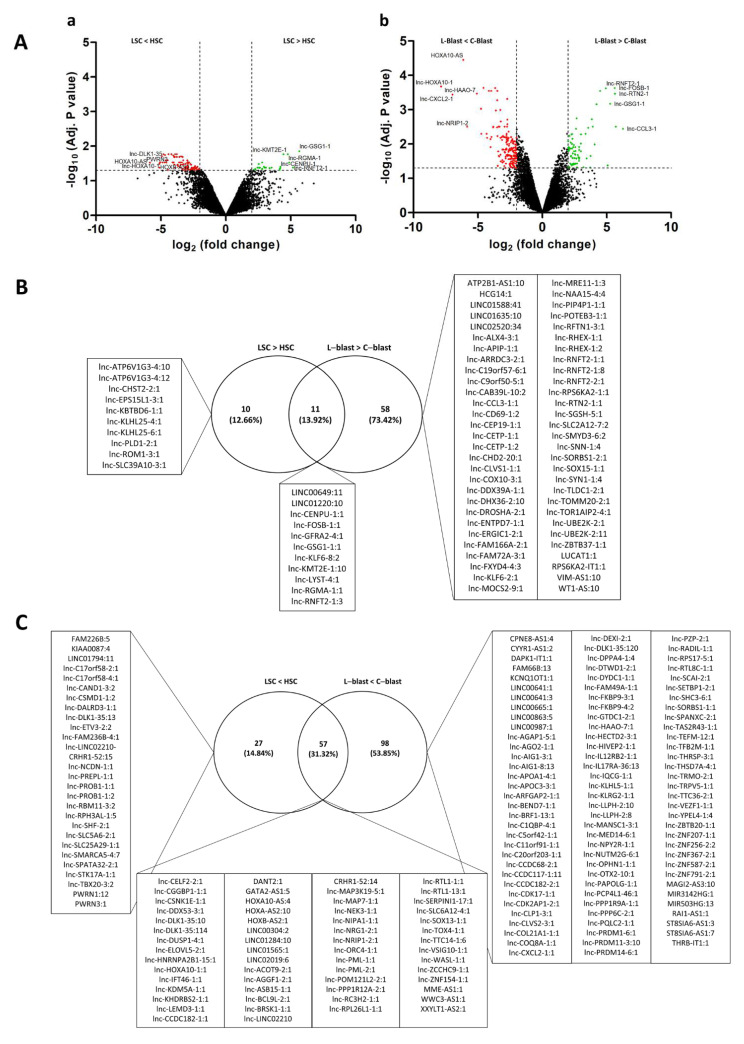
(**A**) lncRNAs are plotted in volcano plots as log2 FC values against −log10 adj. *p* values. (**a**) Volcano plot of the LSC and HSC comparison. (**b**) Volcano plot of the L-blast and C-blast comparison. Thresholds |log2FC| > 2 and log10 adj. *p* ≤ 0.05 are shown as dashed lines, and significantly up- and downregulated lncRNAs are highlighted in green and red, respectively. Top 5 up- and downregulated DE-lncRNAs are indicated. (**B**) Venn diagram of all upregulated lncRNAs (|logFC| ≥ 2, Adj. *p* value < 0.05) in both leukemic fractions compared to their normal counterparts. (**C**) Venn diagram of all downregulated lncRNAs (|logFC| ≥ 2, Adj. *p* value < 0.05) in both leukemic fractions compared to their normal counterparts.

To identify lncRNAs expressed in different pedAML cellular fractions, we performed lncRNA profiling using RNA isolated from CD34+/CD38+ (*n* = 4, L-blast) and CD34+/CD38− (*n* = 3, LSC) sorted cell populations from four pedAML patients. In addition, sorted CD34+/CD38+ (*n* = 3, C-blast) and CD34+/CD38− (*n* = 2, HSC) from CB were profiled to examine lncRNA expression in their normal counterparts. Principal component analysis showed that the different subpopulations cluster correctly and are divergent from the healthy CB controls (Figure 1). From the 30,168 probes with corresponding annotated lncRNA (LNCipedia 5.2 version 2.0), 105 significantly DE-lncRNAs (|log2FC| > 2 and adj. *p* ≤ 0.05) were identified in LSCs compared to HSCs, of which 21 were upregulated and 84 downregulated (Figure 2 and Figure 3Aa, Supplementary Table S2). Comparing L-blasts to C-blasts, 225 significant DE-lncRNAs were uncovered, of which 69 were upregulated and 156 downregulated (Figure 2 and Figure 3Ab, Supplementary Table S3).

To further delineate potential interesting therapeutic targets, a threshold for the absolute expression in the HSC fraction was set to expression values less than seven, corresponding to the black corner expression plus one and ensuring low or no expression in the HSC fraction. This resulted in nine upregulated DE-lncRNAs, of which three were unique to the LSC-HSC differential expression analysis (Figure 2, Table 2). Although the majority of the nine upregulated DE-lncRNAs could not be examined in the LncScape database (www.lncscape.de, accessed on 24 March 2022), probably as a consequence of the rapidly evolving knowledge on and discovery of novel lncRNAs, we could observe low HSC expression for *lnc-GSG1-1*, in agreement with our results. In the L-blast fraction, 37 upregulated DE-lncRNAs remained, of which 31 were assigned to the L-blast versus C-blast comparison (Figure 2).

### 3.2. Identification of Potential Functional Networks

**Figure 4:**
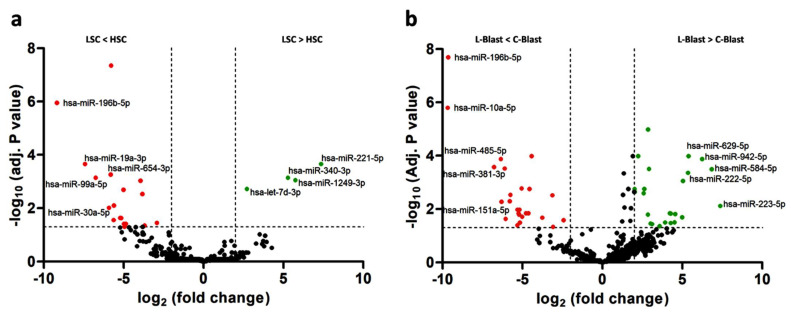
MiRNAs are plotted in volcano plots as log 2 FC values against −log10 adj. *p* values. (**a**) Volcano plot of miRNAs in LSCs compared to HSCs. (**b**) Volcano plot of miRNAs for L-blasts compared to C-blasts. Threshold |log2FC| > 2 and −log10 adj. *p* ≤ 0.05 are shown as dashed lines, and significantly up- and downregulated miRNAs are highlighted in green and red, respectively. Top 5 up- and downregulated DE-miRNAs are indicated.

In order to assess the sponging activity of lncRNAs in pedAML, we performed a small RNA sequencing on the samples used for the lncRNA expression profiling. A total of 627 miRNAs were examined for differential expression analysis, and 22 were found to be significantly differentially expressed (|logFC| > 2 and Adj. *p* value ≤ 0.05) in the LSCs compared to the HSCs, with 4 upregulated and 18 downregulated (Figure 4a, Supplementary Table S4). Comparison of L-blasts to C-blasts revealed 60 significantly differentially expressed miRNAs, with 25 upregulated and 26 downregulated (Figure 4b, Supplementary Table S5).

**Figure 5:**
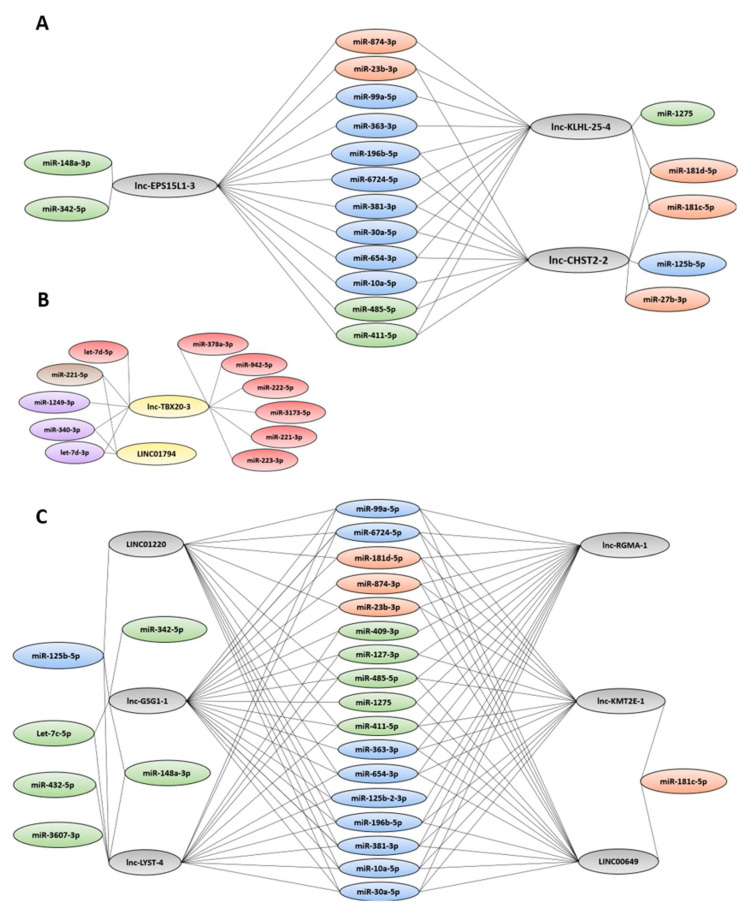
Network of up- (**A**) and downregulated (**B**) LSC DE-lncRNAs and shared DE-lncRNAs (**C**) with anti-correlated miRNAs. Up- and downregulated DE-lncRNAs are represented in grey and yellow, respectively. The anti-correlated miRNAs that are differentially downregulated (|logFC| ≤ 2, Adj. *p* value ≤ 0.05) in the LSCs, the L-blasts, and the LSCs as L-blasts, in this respective order, are visualized in orange, green, and blue. Upregulated (logFC ≥ 2, Adj. *p* value ≤ 0.05) DE-miRNAs in the LSCs, L-blasts, and LSCs as L-blasts are visualized in purple, red, and brown respectively.

Next, the sets of up- and downregulated DE-lncRNAs in the LSCs (unique or shared with the L-blast comparison) (Figure 2) were examined for anti-correlated DE-miRNAs (|ρ| > 0.7). Most of the anti-correlated DE-miRNAs could be found in both the upregulated LSC DE-lncRNA and shared DE-lncRNA groups (Figure 5A,C). A set of seven miRNAs (*miR-196b-5p*, *miR-381-3p*, *miR-411-5p*, *miR-485-5p*, *miR-654-3p*, *miR-6724-5p,* and *miR-30a-5p*) were anti-correlated to all upregulated DE-lncRNAs (unique to LSCs or shared with L-blasts). Interestingly, *miR-27b-3p* was anti-correlated to a single DE-lncRNA, *lnc-CHST2-2*. Moreover, both *miR-27b-3p* and *lnc-CHST2-2* were uniquely differentially expressed in the LSC fraction, and *miR-342-5p* was only anti-correlated to *lnc-EPS15L1-3* (unique) and *lnc-GSG1-1* (shared). Narrowed to the downregulated DE-lncRNAs, 12 miRNAs were identified, with only *miR-221-5p, miR-340-3p*, and *let-7d-3p* anti-correlated to *LINC01794* (Figure 5B).

### 3.3. Identification of Potential Functional Relevance of Unique LSC DE-lncRNAs and Shared DE-lncRNAs

To predict the functional relevance of the DE-lncRNAs, a pre-ranked GSEA was performed on a gene set of correlated protein-coding genes with a Pearson correlation coefficient of >0.7. The focus was narrowed to the unique DE-lncRNAs in the LSCs and the shared DE-lncRNAs. Involvement in various cancer pathways was examined using the hallmark gene set (MSigDB) to identify potential functional relevance in cancer development.

**Figure 6:**
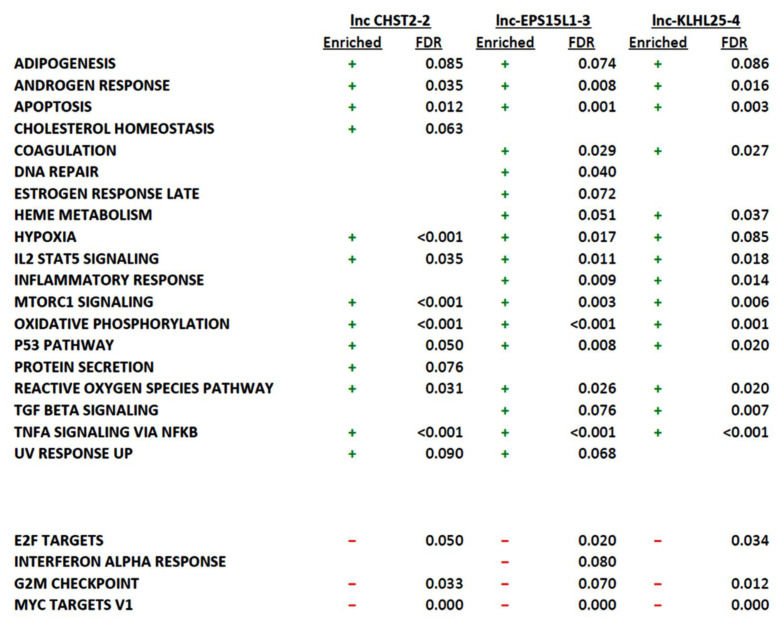
Overview of the pre-ranked GSEA of protein (anti-)correlated with the three unique LSC DE-lncRNAs, showing their involvement in different hallmark pathways (MSigDB) together with their respective FDR values. Plus and minus signs refer to enriched pathways and downregulated pathways within the respective lncRNAs.

For the unique DE-lncRNAs in the LSCs, a GSEA of correlated protein-coding genes showed 13 (*lnc-CHST2-2)*, 17 (*lnc-EPS15L1-3)*, and 14 (*lnc-KLHL25-4)* hallmark pathways, with an FDR <10%. Most correlated genes were involved in the pathways of *TNFA-signaling* via *NFKB*, *IL2 STAT5 signaling, P53*, *hypoxia*, and *apoptosis* (Figure 6). For the GSEA of the anti-correlated protein-coding genes, three (*lnc-CHST2-2),* four (*lnc-EPS15L1-3*), and three (*lnc-KLHL25-4*) enriched pathways were found to have an FDR <10% (Figure 6). For the shared DE-lncRNAs, 14 (*lnc-GSG1-1*), 15 (*lnc-RGMA-1*), 17 (*lnc-KMT2E-1*), 21 (*LINC01220*), 16 (*LINC00649*), and 22 (*lnc-LYST-4*) hallmark pathways had an FDR <10% with most correlated protein-coding genes being involved in the *androgen response*, *apoptosis*, *DNA repair*, *IL2-STAT5 signaling*, *MTORC1 signaling*, *oxidative phosphorylation*, and *TNFA-signaling* via *NFKB pathways* (Supplementary Figure S1). An overview of the protein-coding genes involved in the respective pathways enriched for the DE-lncRNAs can be found in Supplementary Tables S6 and S7.

## 4. Discussion

Although the survival of pedAML has steadily increased during the past decade, relapses occur in almost 40% of patients and remain an important cause of death [2]. The further refinement of risk-stratification strategies and the identification of novel therapeutics targeting the LSC fraction, thought to be responsible for relapse, are warranted. The recently increased interest in studying the non-coding part of the human genome has shown the involvement of non-coding actors, including lncRNAs and miRNAs, in the development of solid and hematopoietic cancers, revealing potential cancer-associated targets. In this study, we identified a novel set of DE-lncRNAs and their associated transcriptional network in LSC and L-blast subpopulations of pediatric AML patients.

Although they have not yet been previously described, three lncRNAs (*lnc-CHST2-2*, *lnc-EPS15L1-3*, and *lnc-KLHL25-4*) were found to be uniquely upregulated in the LSC fraction of pedAML patients. Taken together with their low expression in HSCs, these three lncRNAs might be excellent targets for further therapeutic developments using, e.g., antisense oligonucleotides, as previously demonstrated in other cancer types [21,22]. Similarly, two lncRNAs (*LINC01794* and *lnc-TBX20-3*) are uniquely downregulated in the LSCs. These two should be further explored as potential prognostic biomarkers, and uncovering their functional network may lead to new therapeutic opportunities.

Further corroborating our results, *lnc-RNFT2-1* and *lnc-CLVS1-1*, identified as lncRNAs uniquely differentially expressed in the L-blast fraction, have previously been shown to be differentially expressed in CD34+ hematopoietic stem and progenitor cells of patients with MDS compared to healthy donors through micro-array profiling by Liu and colleagues [23]. As MDS patients bear a high risk of developing AML and patients with MDS-AML often have a worse prognosis [24], it will be interesting to further elaborate on the functional roles of *lnc-RNFT2-1* and *lnc-CLVS1-1* in AML and MDS.

Several lncRNAs (*lnc-GSG1-1*, *lnc-RGMA-1*, *lnc-KMT2E-1*, *LINC01220*, *LINC00649*, and *lnc-LYST-4*) were upregulated in LSCs as well as in L-blasts in comparison to their normal counterparts. Interestingly, the knockdown of *LINC01220* in endometrial carcinoma has been shown to inhibit proliferation and induce apoptosis, suggesting its therapeutic potential [25]. In contrast to our findings, *LINC00649* expression was recently shown to be aberrantly low in AML patients [26], although in many other cancer types, high expression of *LINC00649* has been shown to be crucial in driving carcinogenesis [27,28,29]. Functional work will need to further establish the role of *LINC00649* specifically in the context of pedAML.

MicroRNA expression analyses in this study revealed many known and less-studied microRNAs in the context of AML. *MiR-196b-5p*, *miR-654-3p*, and *miR-485-5p* were identified as anti-correlated to all upregulated LSC DE-lncRNAs (unique and shared with L-blasts). Both *miR-196b-5p* and *miR-654-3p* were previously shown to be involved in AML progression, and *miR-485-5p* may have a role in suppression of apoptosis in AML [30,31,32]. *MiR-10a-5p*, anti-correlated to all shared upregulated DE-lncRNAs and two upregulated DE-lncRNAs in the LSCs, is a known prognostic biomarker in FLT3- and NPM1-mutated AML [33,34,35]. Additionally, *miR-23b-3p* anti-correlated to all but one (*lnc-LYST-4*) upregulated DE-lncRNA (unique and shared) has been shown to scavenge cellular reactive oxygen species (ROS) by targeting peroxiredoxin III, suggesting its role in leukemogenesis since high endogenous ROS levels are required for HSC differentiation [36]. Interestingly, *miR-342-5p* anti-correlated to *lnc-EPS15L1-3* and *lnc-GSG1-1* is downregulated in MDS and CML patients [37,38], while *miR-148a-3p* anti-correlated to *lnc-EPS15L1-3*, *lnc-GSG1-1,* and *lnc-LYST-4* as well is believed to have a functional role in the disease progression of AML [39]. In addition, Cheng and colleagues identified *miR-148a-3p* as part of a tumor microenvironmental competitive endogenous RNA network that acted as a robust prognostic predictor of AML [40]. Furthermore, *miR-874-3p* and *miR-363-3p* were anti-correlated to the majority of the upregulated DE-lncRNAs (unique to LSCs and shared with L-blasts). Although *miR-874-3p* was found to be overexpressed in MDS and AML patients, its downregulation by *DANCR* through sponging may contribute to cytarabine resistance in AML by activating autophagy [41,42]. Finally, *miR-363-3p* promotes the development of RUNX1-mutated AML and affects expression of tumor suppressor genes in T-ALL, modulating survival [43,44].

We aimed to predict the functional relevance of the identified DE-lncRNA through a pre-ranked GSEA of (anti-)correlated (|ρ| > 0.7) protein-coding genes. Interestingly, *CD69*, known to be responsible for the self-renewal capacity of LSCs, was enriched in the hallmark pathways *TNFA signaling* via *NFKB*, *apoptosis*, *inflammatory response*, and *interferon-gamma response* for *lnc-EPS15L1-3* and *lnc-KMT2E-1* as well as all shared DE-lncRNAs. However, exact knowledge of the function of *CD69* in AML is lacking [45]. Furthermore, all identified upregulated DE-lncRNAs (shared and unique) were correlated with *PPP1R15A,* which was enriched in the *TNFA signaling* via *NFKB, MTORC1 signaling*, *hypoxia, p53,* and *TGF beta signaling* hallmark pathways. Although it is not linked to hematological malignancies, *PPP1R15A* is part of a hypoxia-related prognostic signature for breast cancer [46]. Moreover, *SLC2A3* was also correlated with all DE-lncRNAs (unique and shared) and was previously shown to be upregulated in the L-blasts in pedAML [19]. *IER3* and *NAMPT*, which correlated with all but two (*lnc-CHST2-2* and *lnc-LYST-4*) of the upregulated DE-lncRNAs, both have a therapeutic link with AML. On the one hand, *IER3* is normally downregulated in AML, and vorinostat increases *IER3* expression to favor cell cycle arrest, differentiation, and apoptosis of malignant cells [47]. On the other hand, *NAMPT* inhibitors can selectively induce the apoptosis of AML stem cells by disrupting lipid homeostasis [48].

As the aim of this study was to find new therapeutic targets by identifying and exploring functional networks in which lncRNAs play a pivotal role, the focus was narrowed to uniquely upregulated LSC DE-lncRNAs and shared upregulated DE-lncRNAs. However, downregulated lncRNAs also have an important role and may emerge as new prognostic markers predicting the outcome of pedAML patients. Although the exploration of downregulated targets is beyond the scope of our study, our data set also provides leads for further research on downregulated lncRNAs.

This study has some limitations, including its design and the limited number of participants. Consequently, the provided data should be interpreted with caution within the framework of generalizability. In addition, we acknowledge that extensive in vitro and in vivo validation of several of the identified targets is needed to fully uncover the potential of the presented data. Nevertheless, here we provide a repository of potential targets that allow us to fulfill the imperative need for alternative therapeutic strategies in pedAML.

## 5. Conclusions

In conclusion, this study provides a unique set of LSC- and L-blast-specific lncRNAs in pedAML and their interactions with miRNAs, ultimately impacting gene expression. The generated data provide a rich source of promising targets, most of which have not been previously described in pedAML, and networks that could serve as anchor points for further functional experimental research.

## Figures and Tables

**Table 1:** Characteristics of four de novo pedAML patients used for the sorting and micro-array profiling of CD34+/CD38+ and CD34+/CD38− cell fractions. Patients were diagnosed in Belgium and classified as standard risk. WT1 overexpression was interpreted in regard to in-house or published (Cilloni et al. 2009) cut-offs.

Variation		Median (Range)	
Age, years		14 (10–15)	
WBC count, ×10^9^		79 (58.1–118)	
Morphological blast count			
	BM%	88 (34–95)	
	PB%	74 (38–78)	
		N	%
Gender			
	F	3	75.0%
	M	1	25.0%
Study protocol			
	DB AML-01	4	100.0%
CNS involvement		0	0.0%
*WT1* overexpression		2	50.0%
Fusion transcript		2	50.0%
CBF leukemia		2	50.0%
	*AML1::ETO* + *C-KIT*^WT^	1	25.0%
	*AML1::ETO* + *C-KIT^MUT^*	0	0.0%
	*CBF::MYH11*	1	25.0%
Gene mutation			
	*NPM1*	0	0.0%
	*CEBPA*	0	0.0%
*FLT3*			
	ITD	2	50.0%
	WT	2	50.0%
Karyotype			
	Normal	1	25.0%
	Abnormal	3	75.0%
FAB classification			
	M0	1	25.0%
	M2	1	25.0%
	M4	2	50.0%

**Table 2:** Overview of the characteristics of the upregulated LSC lncRNA (unique and shared with the L-blast fraction (LNCipedia, version 5.2).

Transcript ID	Location	Strand	Class
Lnc-CHST2-2:1	chr3:143123362-143131893	+	intronic
Lnc-EPS15L1-3:1	chr19:16283359-16324514	−	bidirectional
Lnc-KLHL25-4:1	chr15:85621264-85627689	−	antisense
Lnc-GSG1-1:1	chr12:13196786-13197774	−	antisense
Lnc-RGMA-1:1	chr15:92805770-92808567	−	intronic
Lnc-KMT2E-1:10	chr7:104941063-104962060	+	intergenic
LINC01220:10	chr14:75294441-75296638	+	intergenic
LINC00649:11	chr21:33931554-33977774	+	antisense
Lnc-LYST-4:1	chr1:235826323-235883708	−	intronic

## Data Availability

Raw data from the micro-array can be found under GSE 128103 (release on 9 March 2022). Raw counts of the small RNA-sequencing analysis can be found under GSE 196886 (release on 16 August 2022).

## Supplementary Materials

The following are available online at https://www.mdpi.com/article/10.3390/cancers14092098/s1: Supplemental Material and Methods [49,50,51]; Figure S1: Overview of the pre-ranked gene set enrichment analysis (GSEA) of protein-coding genes (anti-)correlated with the six shared DE-lncRNAs, showing their involvement in different hallmark pathways (MSigDB); Table S1: Overview of monoclonal antibodies used for membrane staining and sorting; Table S2: Differentially expressed (|logFC| ≤ 2, Adj. *p* value ≤ 0.05) lncRNAs in LSCs compared to HSCs; Table S3: Differentially expressed (|logFC| ≤ 2, Adj. *p* value ≤ 0.05) lncRNAs in L-blasts compared to C-blasts; Table S4: Differentially expressed (|logFC| ≤ 2, Adj. *p* value ≤ 0.05) miRNAs in LSCs compared to HSCs; Table S5: Differentially expressed (|logFC| ≤ 2, Adj. *p* value ≤ 0.05) miRNAs in L-blasts compared to C-blasts; Table S6: Enriched pathways with associated protein-coding genes for the unique upregulated DE-lncRNAs in the LSC fraction; Table S7: Enriched pathways with associated protein-coding genes for the shared upregulated DE-lncRNAs in the LSC fraction and the L-blasts.

## References

[1] Rasche M., Zimmermann M., Borschel L., Bourquin J.-P., Dworzak M., Klingebiel T., Lehrnbecher T., Creutzig U., Klusmann J.-H., Reinhardt D. (2018). Successes and Challenges in the Treatment of Pediatric Acute Myeloid Leukemia: A Retrospective Analysis of the AML-BFM Trials from 1987 to 2012. Leukemia.

[2] De Moerloose B., Reedijk A., de Bock G.H., Lammens T., de Haas V., Denys B., Dedeken L., van den Heuvel-Eibrink M.M., Te Loo M., Uyttebroeck A. (2019). Response-Guided Chemotherapy for Pediatric Acute Myeloid Leukemia without Hematopoietic Stem Cell Transplantation in First Complete Remission: Results from Protocol DB AML-01. Pediatr. Blood Cancer.

[3] Zwaan C.M., Kolb E.A., Reinhardt D., Abrahamsson J., Adachi S., Aplenc R., De Bont E.S.J.M., De Moerloose B., Dworzak M., Gibson B.E.S. (2015). Collaborative Efforts Driving Progress in Pediatric Acute Myeloid Leukemia. J. Clin. Oncol..

[4] Depreter B., Weening K.E., Vandepoele K., Essand M., De Moerloose B., Themeli M., Cloos J., Hanekamp D., Moors I., D’hont I. (2020). TARP Is an Immunotherapeutic Target in Acute Myeloid Leukemia Expressed in the Leukemic Stem Cell Compartment. Haematologica.

[5] Depreter B., De Moerloose B., Vandepoele K., Uyttebroeck A., Van Damme A., Denys B., Dedeken L., Dresse M.-F., Van der Werff Ten Bosch J., Hofmans M. (2020). Clinical Significance of TARP Expression in Pediatric Acute Myeloid Leukemia. Hemasphere.

[6] Wang J., Chen S., Xiao W., Li W., Wang L., Yang S., Wang W., Xu L., Liao S., Liu W. (2018). CAR-T Cells Targeting CLL-1 as an Approach to Treat Acute Myeloid Leukemia. J. Hematol. Oncol..

[7] Chapuis A.G., Egan D.N., Bar M., Schmitt T.M., McAfee M.S., Paulson K.G., Voillet V., Gottardo R., Ragnarsson G.B., Bleakley M. (2019). T Cell Receptor Gene Therapy Targeting WT1 Prevents Acute Myeloid Leukemia Relapse Post-Transplant. Nat. Med..

[8] Taussig D.C., Pearce D.J., Simpson C., Rohatiner A.Z., Lister T.A., Kelly G., Luongo J.L., Danet-Desnoyers G.-A.H., Bonnet D. (2005). Hematopoietic Stem Cells Express Multiple Myeloid Markers: Implications for the Origin and Targeted Therapy of Acute Myeloid Leukemia. Blood.

[9] Cai S.F., Levine R.L. (2019). Genetic and Epigenetic Determinants of AML Pathogenesis. Semin. Hematol..

[10] Bill M., Papaioannou D., Karunasiri M., Kohlschmidt J., Pepe F., Walker C.J., Walker A.E., Brannan Z., Pathmanathan A., Zhang X. (2019). Expression and Functional Relevance of Long Non-Coding RNAs in Acute Myeloid Leukemia Stem Cells. Leukemia.

[11] Schwarzer A., Emmrich S., Schmidt F., Beck D., Ng M., Reimer C., Adams F.F., Grasedieck S., Witte D., Käbler S. (2017). The Non-Coding RNA Landscape of Human Hematopoiesis and Leukemia. Nat. Commun..

[12] Bhan A., Soleimani M., Mandal S.S. (2017). Long Noncoding RNA and Cancer: A New Paradigm. Cancer Res..

[13] Choudhari R., Sedano M.J., Harrison A.L., Subramani R., Lin K.Y., Ramos E.I., Lakshmanaswamy R., Gadad S.S. (2020). Long Noncoding RNAs in Cancer: From Discovery to Therapeutic Targets. Adv. Clin. Chem..

[14] Geisler S., Coller J. (2013). RNA in Unexpected Places: Long Non-Coding RNA Functions in Diverse Cellular Contexts. Nat. Rev. Mol. Cell Biol..

[15] Li Q., Wang J. (2020). LncRNA TUG1 Regulates Cell Viability and Death by Regulating MiR-193a-5p/Rab10 Axis in Acute Myeloid Leukemia. OncoTargets Ther..

[16] Zhuang M.-F., Li L.-J., Ma J.-B. (2019). LncRNA HOTTIP Promotes Proliferation and Cell Cycle Progression of Acute Myeloid Leukemia Cells. Eur. Rev. Med. Pharmacol. Sci..

[17] Cheng P., Lu P., Guan J., Zhou Y., Zou L., Yi X., Cheng H. (2020). LncRNA KCNQ1OT1 Controls Cell Proliferation, Differentiation and Apoptosis by Sponging MiR-326 to Regulate c-Myc Expression in Acute Myeloid Leukemia. Neoplasma.

[18] Chang L., Guo R., Yuan Z., Shi H., Zhang D. (2018). LncRNA HOTAIR Regulates CCND1 and CCND2 Expression by Sponging MiR-206 in Ovarian Cancer. Cell Physiol. Biochem..

[19] Depreter B., De Moerloose B., Vandepoele K., Uyttebroeck A., Van Damme A., Terras E., Denys B., Dedeken L., Dresse M.-F., Van der Werff Ten Bosch J. (2021). Deciphering Molecular Heterogeneity in Pediatric AML Using a Cancer vs. Normal Transcriptomic Approach. Pediatr. Res..

[20] Volders P.-J., Anckaert J., Verheggen K., Nuytens J., Martens L., Mestdagh P., Vandesompele J. (2019). LNCipedia 5: Towards a Reference Set of Human Long Non-Coding RNAs. Nucleic Acids Res..

[21] Dewaele S., Delhaye L., De Paepe B., de Bony E.J., De Wilde J., Vanderheyden K., Anckaert J., Yigit N., Nuytens J., Vanden Eynde E. (2021). The Long Non-Coding RNA SAMMSON Is Essential for Uveal Melanoma Cell Survival. Oncogene.

[22] Hofmans M., Lammens T., Depreter B., Wu Y., Erlacher M., Caye A., Cavé H., Flotho C., de Haas V., Niemeyer C.M. (2021). Long Non-Coding RNAs as Novel Therapeutic Targets in Juvenile Myelomonocytic Leukemia. Sci. Rep..

[23] Liu K., Beck D., Thoms J.A.I., Liu L., Zhao W., Pimanda J.E., Zhou X. (2017). Annotating Function to Differentially Expressed LincRNAs in Myelodysplastic Syndrome Using a Network-Based Method. Bioinformatics.

[24] Montalban-Bravo G., Kanagal-Shamanna R., Class C.A., Sasaki K., Ravandi F., Cortes J.E., Daver N., Takahashi K., Short N.J., DiNardo C.D. (2020). Outcomes of Acute Myeloid Leukemia with Myelodysplasia Related Changes Depend on Diagnostic Criteria and Therapy. Am. J. Hematol..

[25] Li Y., Kong C., Wu C., Wang Y., Xu B., Liang S., Ying X. (2019). Knocking down of LINC01220 Inhibits Proliferation and Induces Apoptosis of Endometrial Carcinoma through Silencing MAPK11. Biosci. Rep..

[26] Guo C., Gao Y.-Y., Ju Q.-Q., Zhang C.-X., Gong M., Li Z.-L. (2020). LINC00649 Underexpression Is an Adverse Prognostic Marker in Acute Myeloid Leukemia. BMC Cancer.

[27] Chen X., Chen S. (2021). LINC00649 Promotes Bladder Cancer Malignant Progression by Regulating the MiR-15a-5p/HMGA1 Axis. Oncol. Rep..

[28] Wang H., Di X., Bi Y., Sun S., Wang T. (2021). Long Non-Coding RNA LINC00649 Regulates YES-Associated Protein 1 (YAP1)/Hippo Pathway to Accelerate Gastric Cancer (GC) Progression via Sequestering MiR-16-5p. Bioengineered.

[29] Ye G., Guo L., Xing Y., Sun W., Yuan M. (2019). Identification of Prognostic Biomarkers of Prostate Cancer with Long Non-Coding RNA-Mediated Competitive Endogenous RNA Network. Exp. Ther. Med..

[30] Hu Q., Gu Y., Chen S., Tian Y., Yang S. (2020). Hsa_circ_0079480 Promotes Tumor Progression in Acute Myeloid Leukemia via MiR-654-3p/HDGF Axis. Aging.

[31] Zhang F., Li Q., Zhu K., Zhu J., Li J., Yuan Y., Zhang P., Zhou L., Liu L. (2020). LncRNA LINC00265/MiR-485-5p/IRF2-Mediated Autophagy Suppresses Apoptosis in Acute Myeloid Leukemia Cells. Am. J. Transl. Res..

[32] Liu W., Cheng F. (2021). Circular RNA CircCRKL Inhibits the Proliferation of Acute Myeloid Leukemia Cells via the MiR-196a-5p/MiR-196b-5p/P27 Axis. Bioengineered.

[33] Chen S., Chen Y., Zhu Z., Tan H., Lu J., Qin P., Xu L. (2020). Identification of the Key Genes and MicroRNAs in Adult Acute Myeloid Leukemia with FLT3 Mutation by Bioinformatics Analysis. Int. J. Med. Sci..

[34] Havelange V., Ranganathan P., Geyer S., Nicolet D., Huang X., Yu X., Volinia S., Kornblau S.M., Andreeff M., Croce C.M. (2014). Implications of the MiR-10 Family in Chemotherapy Response of NPM1-Mutated AML. Blood.

[35] Zhi F., Cao X., Xie X., Wang B., Dong W., Gu W., Ling Y., Wang R., Yang Y., Liu Y. (2013). Identification of Circulating MicroRNAs as Potential Biomarkers for Detecting Acute Myeloid Leukemia. PLoS ONE.

[36] Jiang W., Min J., Sui X., Qian Y., Liu Y., Liu Z., Zhou H., Li X., Gong Y. (2015). MicroRNA-26a-5p and MicroRNA-23b-3p up-Regulate Peroxiredoxin III in Acute Myeloid Leukemia. Leuk. Lymphoma.

[37] Choi Y., Hur E.-H., Moon J.H., Goo B.-K., Choi D.R., Lee J.-H. (2019). Expression and Prognostic Significance of MicroRNAs in Korean Patients with Myelodysplastic Syndrome. Korean J. Intern. Med..

[38] Wu Y.-Y., Lai H.-F., Huang T.-C., Chen Y.-G., Ye R.-H., Chang P.-Y., Lai S.-W., Chen Y.-C., Lee C.-H., Liu W.-N. (2021). Aberrantly Reduced Expression of MiR-342-5p Contributes to CCND1-Associated Chronic Myeloid Leukemia Progression and Imatinib Resistance. Cell Death Dis..

[39] Zhou H., Jia X., Yang F., Shi P. (2021). MiR-148a-3p Suppresses the Progression of Acute Myeloid Leukemia via Targeting Cyclin-Dependent Kinase 6 (CDK6). Bioengineered.

[40] Cheng Y., Wang X., Qi P., Liu C., Wang S., Wan Q., Liu Y., Su Y., Jin L., Liu Y. (2021). Tumor Microenvironmental Competitive Endogenous RNA Network and Immune Cells Act as Robust Prognostic Predictor of Acute Myeloid Leukemia. Front. Oncol..

[41] Ozdogan H., Gur Dedeoglu B., Oztemur Islakoglu Y., Aydos A., Kose S., Atalay A., Yegin Z.A., Avcu F., Uckan Cetinkaya D., Ilhan O. (2017). DICER1 Gene and MiRNA Dysregulation in Mesenchymal Stem Cells of Patients with Myelodysplastic Syndrome and Acute Myeloblastic Leukemia. Leuk. Res..

[42] Zhang H., Liu L., Chen L., Liu H., Ren S., Tao Y. (2021). Long Noncoding RNA DANCR Confers Cytarabine Resistance in Acute Myeloid Leukemia by Activating Autophagy via the MiR-874-3P/ATG16L1 Axis. Mol. Oncol..

[43] Chen Y., Chen S., Lu J., Yuan D., He L., Qin P., Tan H., Xu L. (2021). MicroRNA-363-3p Promote the Development of Acute Myeloid Leukemia with RUNX1 Mutation by Targeting SPRYD4 and FNDC3B. Medicine.

[44] Drobna M., Szarzyńska B., Jaksik R., Sędek Ł., Kuchmiy A., Taghon T., Van Vlierberghe P., Szczepański T., Witt M., Dawidowska M. (2020). Hsa-MiR-20b-5p and Hsa-MiR-363-3p Affect Expression of PTEN and BIM Tumor Suppressor Genes and Modulate Survival of T-ALL Cells In Vitro. Cells.

[45] Sachs K., Sarver A.L., Noble-Orcutt K.E., LaRue R.S., Antony M.L., Chang D., Lee Y., Navis C.M., Hillesheim A.L., Nykaza I.R. (2020). Single-Cell Gene Expression Analyses Reveal Distinct Self-Renewing and Proliferating Subsets in the Leukemia Stem Cell Compartment in Acute Myeloid Leukemia. Cancer Res..

[46] Wang J., Wang Y., Xing P., Liu Q., Zhang C., Sui Y., Wu C. (2020). Development and Validation of a Hypoxia-Related Prognostic Signature for Breast Cancer. Oncol. Lett..

[47] Silva G., Cardoso B.A., Belo H., Almeida A.M. (2013). Vorinostat Induces Apoptosis and Differentiation in Myeloid Malignancies: Genetic and Molecular Mechanisms. PLoS ONE.

[48] Subedi A., Liu Q., Ayyathan D.M., Sharon D., Cathelin S., Hosseini M., Xu C., Voisin V., Bader G.D., D’Alessandro A. (2021). Nicotinamide Phosphoribosyltransferase Inhibitors Selectively Induce Apoptosis of AML Stem Cells by Disrupting Lipid Homeostasis. Cell Stem Cell.

[49] Kersten B., Valkering M., Wouters R., van Amerongen R., Hanekamp D., Kwidama Z., Valk P., Ossenkoppele G., Zeijlemaker W., Kaspers G. (2016). CD45RA, a specific marker for leukaemia stem cell sub-populations in acute myeloid leukaemia. Br. J. Haematol..

[50] Zeijlemaker W., Kelder A., Wouters R., Valk P.J., Witte B.I., Cloos J., Ossenkoppele G.J., Schuurhuis G.J. (2015). Absence of leukaemic CD34 cells in acute myeloid leukaemia is of high prognostic value: A longstanding controversy deciphered. Br. J. Haematol..

[51] Zeijlemaker W., Kelder A., Oussoren-Brockhoff Y.J., Scholten W.J., Snel A.N., Veldhuizen D., Cloos J., Ossenkoppele G.J., Schuurhuis G.J. (2016). A simple one-tube assay for immunophenotypical quantification of leukemic stem cells in acute myeloid leukemia. Leukemia.

