# Supplementary material for: Deciphering the Non-Coding RNA Landscape of Pediatric Acute Myeloid Leukemia
Source: Cancers (Basel). 2022 Apr 22;14(9):2098. doi: 10.3390/cancers14092098 (PMC9100904; doi:10.3390/cancers14092098)
Supplement: Supplementary file 1 [file cancers-14-02098-s001.zip › Supplementary.pdf]

Overview Supplementary

Supplemental Material and Methods 2

- Sorting

- Micro-array experimental settings

Supplemental Figure 3

- Figure S1. Overview of the pre-ranked gene set enrichment analysis (GSEA) of protein-coding genes (anti-)correlated with the six shared DE-lncRNAs, showing their involvement in different hallmark pathways (MSigDB).

Supplemental References 4

Supplemental Tables (Excel file: *SupplementalTables.cvs*)

- Table S1. Overview of monoclonal antibodies used for membrane staining and sorting

- Table S2. Differential expressed (|logFC|>2 and Adj.P.Val ≤ 0.05) lncRNAs in LSC compared to HSC

- Table S3. Differential expressed (|logFC|>2 and Adj.P.Val ≤ 0.05) lncRNAs in L-blast compared to C-blast

- Table S4. Differential expressed (|logFC|>2 and Adj.P.Val ≤ 0.05) miRNAs in LSC compared to HSC

- Table S5. Differential expressed (|logFC|>2 and Adj.P.Val ≤ 0.05) miRNAs in L-blast compared to C-blast

- Table S6. Enriched pathways with associated protein-coding genes for the unique upregulated DE-lncRNAs in the LSC fraction

- Table S7. Enriched pathways with associated protein-coding genes for the shared upregulated DE-lncRNAs in the LSC fraction and L-blast

Supplemental Material and Methods

Sorting

CD34+/CD38+ (n=4) and CD34+/CD38- (n=3) cell fractions, and lymphocytes (n=4), were sorted from four de novo pedAML patients and used for profiling. RNA yield for the CD34+/CD38- fraction (LSC) of pedAML4 was insufficient for micro-array profiling. As control, CD34+/CD38+ (n=3) and CD34+/CD38- (n=2) cells were sorted from cord blood (CB). The patient samples were a priori cryopreserved and thawed by short incubation in a 42°C pre-heated water bath, followed by 30 min incubation at room temperature (RT) in 20 mL RPMI with 20% FCS, 200 µL DNase I (1 mg/mL, grade II bovine pancreas) and 200 µL MgCl2 (1 M) (Sigma-Aldrich). After incubation, cells were spinoculated (10 min, 400 rpm) and washed with 15 mL RPMI/20% FCS. The CB samples were sorted using fresh material.

Freshly collected and thawed mononuclear cells (MNCs) were spinoculated (5 min, 1500 rpm). Monoclonal antibodies were added to the cell pellet (mAb, Table S1). After 20 min incubation in the dark at RT, cell pellets were washed with PBS+2% BSA. Next, labeled cells were resuspended in 50% RPMI/50%FCS medium and sorted on a FACSAria III with red, blue, and ultraviolet lasers (BD Biosciences).

All scatters were devoid of cell debris and doublets based on propidium iodide exclusion and FSC-H vs FSC-A plots, respectively. The immature myeloid compartment was defined by CD34, CD45 and scatter properties. CD34-positive cases were identified as those with >1% of CD34+ blasts in the leukemic CD45low/SSClow compartment [1, 2]. CD34+/CD38+ blasts and CD34+/CD38- stem cells were gated as previously described [3]. Lymphocytes and fluorescence-minus-one (FMO) controls were used to determine expression cut-offs for CD38 and LSC aberrant markers. Delineated cell populations were backgated on FSC-A/SSC-A and CD45/SSC-A scatter plots to exclude non-specific events and assure homogeneous scatters.

Micro-array experimental settings

Total RNA was extracted from sorted cells, resuspended in TRIzol, using the miRNeasy Mini Kit (Qiagen) in combination with on-column DNase I digestion (RNase-Free DNase set, Qiagen) according to manufacturer's instructions. RNA quality and concentrations were measured by Agilent 2100 Bioanalyzer (Agilent) and Qubit (ThermoFisher Scientific), respectively. Mean RNA integrity number of all sorted fractions was 9.3 (range 8.6 – 9.9). Three LSC and four L-blast fractions from pedAML patients were profiled. The LSC fraction for one pedAML patient is lacking, as the RNA fraction experienced a technical issue during profiling. Two 2 HSC and three C-blast fractions from healthy controls were profiled. The RNA yield from one HSC fraction appeared to be too low during the experiment, and was therefore excluded.

Profiling was performed by Biogazelle using a custom 8x60K human Gene expression micro-array (Agilent), containing probes for 33178 human protein-coding genes. To this end, 20 ng RNA was preamplified using the Complete Whole Transcriptome Amplification Kit (Sigma-Aldrich). Amplified RNA was subsequently labelled using the Genomic DNA ULS Labeling Kit (Agilent) and hybridized to the array in combination with CGH blocking to reduce background signaling. Micro-arrays were analyzed using an Agilent micro-array scanner and Feature Extraction software (v12.0). Probe intensities were background subtracted, quantile-normalized and log2-based probe intensities were calculated. A target was present if the log2 expression value exceeded the cut-off set at 6.75, based on the dark corner control probe value plus 1. Raw micro-array data are available under the accession number GSE 128103 (released on March 9, 2022).

Data processing of the GSE 128103 dataset was performed in R using the limma package (R Bioconductor). If a gene was represented by multiple probes, the highest expression was selected. Differential expression analyses were performed between LSC and HSC, and L-blast and C-blast, using the limma package (R Bioconductor).

Supplemental Figure

	lnc-GSG1-1		lnc-KMT2E-1		lnc-RGMA-1		LINC00649		LINC01220		lnc-LYST-4	
	Enriched	FDR	Enriched	FDR	Enriched	FDR	Enriched	FDR	Enriched	FDR	Enriched	FDR
ADIPOGENESIS			+	0.031	+	0.014	+	0.007	+	0.015	+	0.043
ANDROGEN RESPONSE	+	0.003	+	0.006	+	0.006	+	0.020	+	0.013	+	0.006
APOPTOSIS	+	0.002	+	0.001	+	<0.001	+	0.006	+	0.002	+	0.002
CHOLESTEROL HOMEOSTASIS									+	0.045	+	0.039
COAGULATION	+	0.049	+	0.083								
DNA REPAIR	+	0.034	+	0.029	+	0.066	+	0.005	+	0.007	+	0.022
ESTROGEN RESPONSE LATE											+	0.04
FATTY ACID METABOLISM					+	0.021			+	0.077		
GLYCOLYSIS											+	0.048
HEME METABOLISM			+	0.082					+	0.028		
HYPOXIA	+	0.001	+	0.007	+	0.002	+	<0.001	+	<0.001	+	<0.001
IL2 STAT5 SIGNALING	+	0.011	+	0.015	+	0.026	+	0.014	+	0.006	+	0.003
INFLAMMATORY RESPONSE	+	0.012	+	0.006			+	0.007	+	0.010		
INTERFERON GAMMA RESPONSE			+	0.085			+	0.054			+	0.052
MTORC1 SIGNALING	+	<0.001	+	<0.001	+	<0.001	+	<0.001	+	<0.001	+	<0.001
MYC TARGETS V1					+	0.062			+	0.014	+	<0.001
OXIDATIVE PHOSPHORYLATION	+	<0.001	+	<0.001	+	<0.001	+	<0.001	+	<0.001	+	<0.001
P53 PATHWAY	+	0.004	+	0.007	+	0.011	+	0.039	+	0.022	+	0.062
PI3K AKT MTOR SIGNALING											+	0.065
PROTEIN SECRETION									+	0.074	+	0.031
REACTIVE OXYGEN SPECIES PATHWAY	+	0.004	+	0.016	+	0.012	+	0.007	+	0.006	+	0.013
TGF BETA SIGNALING	+	0.072	+	0.023	+	0.022	+	0.051	+	0.009	+	0.037
TNFA SIGNALING VIA NFKB	+	<0.001	+	<0.001	+	<0.001	+	<0.001	+	<0.001	+	<0.001
UNFOLDED PROTEIN RESPONSE									+	0.047	+	0.044
UV RESPONSE UP	+	0.036	+	0.027	+	0.005	+	0.015	+	0.005	+	0.006
ALLOGRAFT REJECTION	–	0.094										
APOPTOSIS					–	0.091						
DNA REPAIR	–	0.006	–	0.020	–	0.050			–	0.039		
E2F TARGETS			–	0.020	–	0.042	–	0.009	–	0.035	–	0.094
G2M CHECKPOINT	–	0.001	–	0.023	–	0.002	–	<0.001	–	0.001	–	0.014
MTORC1 SIGNALING					–	0.054	–	0.089				
MYC TARGETS V1	–	<0.001	–	<0.001	–	<0.001	–	<0.001	–	<0.001	–	<0.001
NOTCH SIGNALING					–	0.088						

Figure S1. Overview of the pre-ranked gene set enrichment analysis (GSEA) of protein-coding genes (anti-)correlated with the 6 shared DE-lncRNAs, showing their involvement in different hallmark pathways (MSigDB).

Supplemental references

1. Kersten, B.; Valkering, M.; Wouters, R.; van Amerongen, R.; Hanekamp, D.; Kwidama, Z.; Valk, P.; Ossenkoppele, G.; Zeijlemaker, W.; Kaspers, G.; et al. CD45RA, a specific marker for leukaemia stem cell sub-populations in acute myeloid leukaemia. *Br. J. Haematol.* **2016**, *173*, 219–235.
2. Zeijlemaker, W.; Kelder, A.; Wouters, R.; Valk, P.J.; Witte, B.I.; Cloos, J.; Ossenkoppele, G.J.; Schuurhuis, G.J. Absence of leukaemic CD34 cells in acute myeloid leukaemia is of high prognostic value: a longstanding controversy deciphered. *Br. J. Haematol.* **2015**, *171*, 227–238.
3. Zeijlemaker, W.; Kelder, A.; Oussoren-Brockhoff, Y.J.; Scholten, W.J.; Snel, A.N.; Veldhuizen, D.; Cloos, J.; Ossenkoppele, G.J.; Schuurhuis, G.J. A simple one-tube assay for immunophenotypical quantification of leukemic stem cells in acute myeloid leukemia. *Leukemia* **2016**, *30*, 439–446.
